# Epidemic Models of Contact Tracing: Systematic Review of Transmission Studies of Severe Acute Respiratory Syndrome and Middle East Respiratory Syndrome

**DOI:** 10.1016/j.csbj.2019.01.003

**Published:** 2019-01-26

**Authors:** Kin On Kwok, Arthur Tang, Vivian W.I. Wei, Woo Hyun Park, Eng Kiong Yeoh, Steven Riley

**Affiliations:** a The Jockey Club School of Public Health and Primary Care, The Chinese University of Hong Kong, Hong Kong Special Administrative Region, China; b Stanley Ho Centre for Emerging Infectious Diseases, The Chinese University of Hong Kong, Shatin, Hong Kong Special Administrative Region, China; c Shenzhen Research Institute of The Chinese University of Hong Kong, Shenzhen, China; d Department of Software, Sungkyunkwan University, South Korea; e Department of Electrical and Computer Engineering, Sungkyunkwan University, South Korea; f MRC Centre for Outbreak Analysis and Modelling, Department for Infectious Disease Epidemiology, Imperial College London, United Kingdom

**Keywords:** Contact Tracing, Coronavirus Epidemics, Transmission Modelling, SARS, MERS, Co-V, Coronavirus, MERS, Middle East Respiratory Syndrome, R_0_, Basic reproduction number, SARS, Severe Acute Respiratory Syndrome, SEIR, Susceptible Exposed Infectious Recovered, WHO, World Health Organization

## Abstract

The emergence and reemergence of coronavirus epidemics sparked renewed concerns from global epidemiology researchers and public health administrators. Mathematical models that represented how contact tracing and follow-up may control Severe Acute Respiratory Syndrome (SARS) and Middle East Respiratory Syndrome (MERS) transmissions were developed for evaluating different infection control interventions, estimating likely number of infections as well as facilitating understanding of their likely epidemiology. We reviewed mathematical models for contact tracing and follow-up control measures of SARS and MERS transmission. Model characteristics, epidemiological parameters and intervention parameters used in the mathematical models from seven studies were summarized. A major concern identified in future epidemics is whether public health administrators can collect all the required data for building epidemiological models in a short period of time during the early phase of an outbreak. Also, currently available models do not explicitly model constrained resources. We urge for closed-loop communication between public health administrators and modelling researchers to come up with guidelines to delineate the collection of the required data in the midst of an outbreak and the inclusion of additional logistical details in future similar models.

## 1 Introduction

In the 21st century, there were three large-scale outbreaks in human populations caused by emerging coronaviruses (Co-Vs): (i) Severe Acute Respiratory Syndrome (SARS) outbreak in 2003; (ii) Middle East Respiratory Syndrome (MERS) outbreak in 2012 primarily in the Middle East Saudi Arabian Peninsula region; and (iii) MERS outbreak in 2015 primarily in South Korea. Since their emergence, World Health Organization (WHO) had been notified of more than 8000 confirmed cases of SARS in 26 countries [1] and more than 2200 confirmed cases of MERS in 27 countries [2].

SARS and MERS were both considered as “fast-course” infectious diseases given their relatively short infectious period. However, overall transmission potential for MERS is lower compared to SARS [3] and its outbreaks have been contained with much lower cumulative numbers of infected individuals than was the case for SARS. Prior studies showed that human-to-human transmission of SARS occurred via close contact and respiratory droplets [4], while that of MERS occurred via close contact only [5]. A large proportion of MERS cases were clustered in healthcare settings, most of which were contributed by unprotected close contact between healthcare workers and infected MERS patients. Previous studies have also highlighted the transmission heterogeneity between SARS and MERS. Higher transmission heterogeneity for MERS in the distribution of secondary cases than SARS highlighted the largest outbreak of MERS with sharper incidence peaks [3]. The majority of SARS cases occurred among healthcare workers; while substantial number of MERS cases were patients [3], with most MERS severe cases and mortality being individuals with comorbidities [6]. On the other hand, there were similarities between the two Co-Vs. Diseases caused by them were deadly, with a mortality rate of 29.8% for MERS [7] and 7% for SARS [8].

Contact tracing, the identification and follow-up of individuals who have had contacts with infectious individuals, is a critical process to ensure the best possible chance of control and the longest possible time to local take-off [9]. Contact tracing and follow-up control measures such as quarantine and isolation were crucially important during the SARS outbreak in 2003 [10], the Ebola outbreak in Africa in 2014 [11], as well as its part in the eradication of smallpox [12]. With current advances in vaccine development technologies, the role of contact tracing and follow-up control measures in the initial stage of an epidemic becomes especially important. For some novel pathogens such as pandemic influenza, cutting-edge technology may shorten the time needed for vaccine development after initial isolation, bridging shorter gaps between epidemic emergence and vaccine availability [13]. Should such improvements in vaccine development occur, the potential marginal benefits of improving contact tracing processes will be substantial.

Mathematical models were developed to study the dynamics of SARS and MERS transmissions. Models that explicitly represented how contact tracing and follow-up control measures affected the epidemic dynamics were useful for evaluating different infection control interventions, evaluating burden of infection as well as facilitating further understanding of their epidemiology. Such models have direct utility in planning for future outbreaks of coronaviruses: they can be used to estimate the scale of resources required to conduct effective contact tracing.

In light of this, we conducted a systematic review of mathematical models for contact tracing and follow-up control measures of SARS and MERS transmission. The aims of this review are to (i) provide an overview of contact tracing and follow-up control measures of SARS and MERS transmission gained through mathematical modelling, (ii) to identify future research direction in this area and (iii) to improve future models by addressing current models' deficiencies.

## 2 Methods

To identify articles for the current study, an initial search using the PUBMED/Medline and SCOPUS databases was conducted on 27^th^ October 2018 using the following search terms:

a. (“Contact Tracing” OR “Contact Investigation” OR “Contact Screening”) AND
b. (“model” OR “modelling” OR “modelling”) AND
c. (“SARS” OR “MERS” OR “Middle East Respiratory Syndrome” OR “Severe Acute Respiratory Syndrome”)

### 2.1 Article Selection Criteria

Reviewers used the following selection criteria to include eligible articles:

a. Transmission dynamics modelling studies of SARS or MERS in human populations;
b. Model(s) incorporating contact tracing interventions, case contacts finding, quarantine, contact tracing data, population stratification or that used a heterogeneous contact structure;
c. Model(s) not explicitly discussing contact tracing and follow-up control measures were excluded.
d. Articles not in English were excluded.

### 2.2 Article Selection

**Fig. 1:**
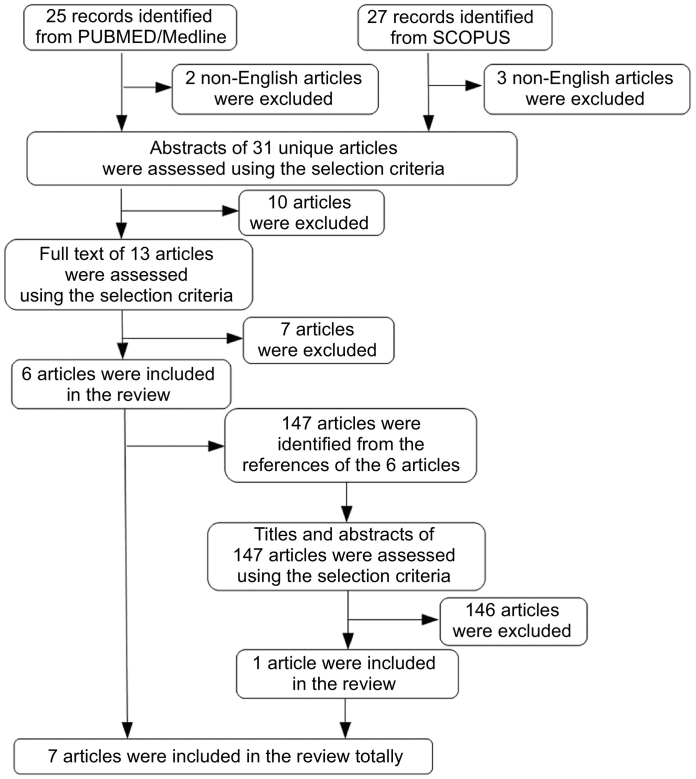
Flow diagram of the selection process.

Two independent reviewers (KOK, AT) screened the titles and abstracts of articles obtained from the initial search and excluded articles that did not fit the selection criteria. The two reviewers then read the full text of the remaining articles, and further excluded articles that did not fit the selection criteria. Finally, reference lists of the included articles were extracted, and the titles and abstracts of these articles from the reference lists were reviewed by the two reviewers based on the article selection criteria. Articles from the extracted reference lists that fit the selection criteria were also included in the current study. The flow diagram of the search process and the result are shown in Fig. 1.

The current systematic review was conducted in accordance with the Preferred Reporting Items for Systematic Reviews and Meta-Analysis: The PRISMA statement [14].

### 2.3 Data Extraction

A standard data extraction form was adopted to extract information from each article, including basic information of the article, characteristics of the study and parameters incorporated in the model.

## 3 Results

Twenty-five articles were identified from the PUBMED/Medline database, and twenty-seven articles were identified from the SCOPUS database with the initial search. Two articles from the PUBMED/Medline database and three articles from the SCOPUS database were excluded because they were not written in English. Sixteen articles were duplicated from the two databases, and thirty-one unique articles were identified from the two databases for assessment of their abstracts using the selection criteria. Eighteen articles were excluded based on their titles and abstracts. Seven articles were further excluded after reading the full text, leaving six articles to be included in the current study. Titles and abstracts of the references of these six articles were further reviewed, and one additional article was included. Therefore, the current study included seven articles for review [[15], [16], [17], [18], [19], [20], [21]].

### 3.1 Characteristics of the Included Studies

Among the seven included studies, four [15,17,19,21] presented the transmission dynamics per individual in an agent-based manner while the other three [16,18,20] modelled from the population perspective and were presented as population-based models. The models by Fraser et al. [19] and Klinkenberg et al. [17] were implemented using agent-based modelling with discrete time simulation. The model by Peak et al. [15] was an agent-based model implemented using Susceptible Exposed Infectious Recovered (SEIR) compartmental branching model. The agent-based model by Becker et al. [21] was a study with household structure. The population-based model by Lloyd-Smith et al. [20] and Feng et al. [16] were implemented using SEIR compartmental model. Chen et al. [18] used Foerster equation-based to describe the population dynamics.

Seven included studies used either (i) population-based modelling, and (ii) agent-based modelling. Population-based modelling is a top-down approach depicting disease dynamics on a system level. Agent-based modelling is a bottom-up approach regarding each individual in the environment as an agent with their own movements and infection states. Population-based models are typically used for analyzing research questions from macroscopic perspectives, whereas agent-based models are good for analyzing microscopic behaviors. Agent-based approach is commonly used to implement heterogeneous and adaptive behaviors. Population-based models are usually less computationally demanding comparing with agent-based models, especially when the number of parameters incorporated in the model is large.

All reviewed studies presented models using SARS as the study example of Co-V; one also presented model studying MERS [15]. Six studies simulated community setting in the models [[15], [16], [17],[19], [20], [21]]; three studies simulated hospital setting in their models [16,18,20]. Six studies assumed homogeneous mixing in the social contact structure [[15], [16], [17], [18], [19], [20]]: the assumption that everyone in the population had the same probability of making social contact with others. One study assumed heterogeneous social contact mixing between school attendees and non-school attendees [21]. All studies considered single-step tracing of contacts directly exposed to the infectious individuals. One study also considered interactive tracing of contacts directly and indirectly exposed to the infectious individuals [17].Characteristics of the seven included studies are summarized in Table 1.

**Table 1:** Summary of characteristics of the reviewed studies.

Author	Lloyd Smith et al.	Fraser et al.	Becker et al.	Chen et al.	Klinkenberg et al.	Feng et al.	Peak et al.
Year	2003	2004	2005	2006	2006	2009	2017
Model type	Population based: Stochastic SEIR compartmental model	Agent based: Discrete time simulation model	Agent based: Branching process with household level transmission model	Population based: Von Foerster equation-based control model	Agent based: Discrete time simulation model	Population based: SEIR compartmental model	Agent based: SEIR compartmental branching model
Co-V to be studied	SARS	SARS	SARS	SARS	SARS	SARS	MERS and SARS
Setting	Community and its hospital	Community	Community of households	Hospital	Community	Community and hospital	Community
Social contact structure	Homogeneous mixing	Homogeneous mixing	Heterogeneous mixing between two groups: school attendees and non-school attendees	Homogeneous mixing	Homogeneous mixing	Homogeneous mixing	Homogeneous mixing
Types of tracing	Single-step	Single-step	Single-step	Single-step	Single-step or Interactive	Single-step	Single-step
Self-reported limitations	1. Superspreading event was not considered in the model. 2. Non-infected patients in hospitals was not considered in the model.	Overestimation in contact tracing efficacy due to failure of identifying correlation structure between diseases generation by contact tracing.	The effect of different interventions on infection dynamics was not considered.	Transmission heterogeneity such as different social contact mixing was not considered in the model.	1. The model only considers transmission before tracing or isolation. 2. The model does not incorporate both hospital and community settings.	1. Medical consultation seeking rate and diagnosis probability were combined. 2. Quarantined individuals were assumed to spend half of their incubation period at large.	The study focused on early stage of the outbreak.

### 3.2 Key Input Epidemiological Parameters

There was considerable variation in the assumptions made about key epidemiological parameters: the basic reproduction number (R_0_), incubation period, latent period and infectious period. Key epidemiological parameters used in the seven included studies are summarized in Table 2.

**Table 2:** Summary of key epidemiological parameters used in the reviewed studies.

Author	Lloyd Smith et al.	Fraser et al.	Becker et al.	Chen et al.	Klinkenberg et al.	Feng et al.	Peak et al.
Year	2003	2004	2005	2006	2006	2009	2017
a) Basic reproduction number (Ro)	1.5–5 Ro of 3 was focused	2–4	6	0.25–5.31	1.5, 2 and 3	Not an input parameter	2.9 for SARS 0.95 for MERS
b) Incubation period	Gamma distribution	Gamma distribution with mean of 4.25 days and variance of 14.25 days	Assumed to be equal to latent period	Exponential distribution	Gamma distribution with 3.81 days	Gamma distribution of incubation period was used for Latin Hypercube sampling	4.01 days for SARS 5.20 days for MERS
c) Latent period	Not included in the model	Not included in the model	6.5 days	Not included in the model	6.81 days	Not included in the model	Represented by the latent offset term which refers to the timing of the latent period relative to the incubation period
d) Infectious period	Gamma distribution	Gamma distribution: low variance gamma distribution with a peak at 9.25 days after infection	Effective infectious period of 9 days	Exponential distribution	Effective infectious period of 3.87 days	Gamma distribution of infectious period was used for Latin Hypercube sampling	Represented by time varying relative infectiousness following triangular distribution

#### 3.2.1 Basic Reproduction Number (R_0_)

R_0_ is the average number of secondary cases caused by one typically infectious individual of an epidemic in a wholly susceptible population [22]. R_0_ used by transmission models were usually estimated based on clinical data, and they varied by the dataset used. The values of R_0_ for SARS used by the seven included studies ranged from 0.25 to 6; and the value of R_0_ for MERS used by Peak et al. was 0.95 [15].

#### 3.2.2 Incubation Period and Latent Period

**Fig. 2:**
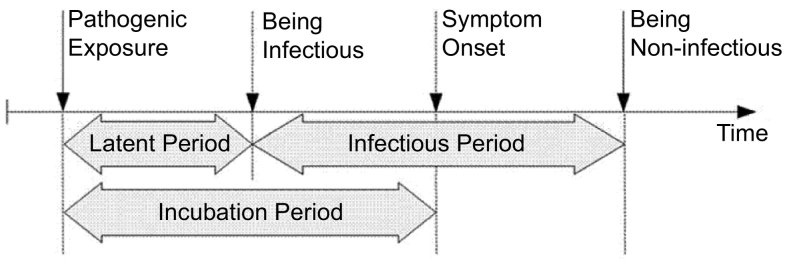
Disease progression periods in the natural history of a disease course. Note that symptom onset can occur before or after being infectious.

Incubation period refers to the time elapsed between pathogenic exposures to symptom onset [23] whereas latent period is the time elapsed between pathogenic exposures to being infectious [24]. Fig. 2 illustrates these two periods in the natural history of a disease course, where symptom onset can occur before or after being infectious. Six studies incorporated incubation period in the models. They characterized the incubation period with three different parametric distributions. Lloyd-Smith et al., Fraser et al., Feng et al. and Klinkenberg et al. assumed gamma distribution [16,17,19,20]. Chen et al. assumed exponential distribution [18]. Peak et al. did not assume any distribution [15].

Three studies incorporated latent period in their models [15,17,21]. Klinkenberg et al. defined latent period relative to detection time by subtracting latent period from the sum of the incubation period and the time lapse from symptom onset to isolation [17]. Becker et al. assumed that symptom onsets occurred prior to infection, and used incubation period from previous literatures as latent period [21]. Peak et al. did not incorporate latent period as a parameter; the latent period offset was introduced as a parameter to measure the timing of the latent period relative to the incubation period [15].

In our two study Co-Vs, SARS had a slightly shorter incubation period than latent period [15,17]. However, a longer incubation period than latent period was observed in MERS with occurrence of symptom onset after being infectious as presented as positive latent period offset in the model by Peak et al. [15].

#### 3.2.3 Infectious Period

The infectious period is the time interval during which the infected individuals could transmit the disease to any susceptible contacts (Fig. 2) Lloyd-Smith et al., Fraser et al. and Feng et al. [16,19,20] assumed gamma-distributed infectious period, while Chen et al. [18] assumed exponentially-distributed infectious period. Peak et al. [15] assumed a triangular distribution using peak infectiousness as a parameter of the distribution. Klinkenberg et al. and Becker et al. [17,21] used a similar term called “effective infectious period”, defined as the time lapse from being infectious to isolation Klinkenberg et al. [17] and Becker et al. [21] both assumed a constant effective infectious period adopted from previous literature and WHO data respectively.

### 3.3 Intervention Parameters

The process of contact tracing sits on top of the transmission model and is governed by its own parameters within these models. Intervention parameters used in the seven reviewed studies are summarized in Table 3.

**Table 3:** Summary of intervention parameters of the reviewed studies.

Author	Lloyd Smith et al.	Fraser et al.	Becker et al.	Chen et al.	Klinkenberg et al.	Feng et al.	Peak et al.
Year	2003	2004	2005	2006	2006	2009	2017
a) Successful tracing ratio	Not included in the model	Not included in the model	Assuming 100% traced on all household members of the primary household infective and a proportion of 50% of between household contact of an infective	Yes; Not explicitly included in the model but reflected in contact tracing efficacy	Yes; named as probability of contact being traced	Not included in the model	Yes; named as proportion of contact traced;
b) Asymptomatic infection ratio	0–10%	0–11%	Not included in the model	0.01–11%	Not included in the model	Yes; reflected in infection rates contributed by exposed individuals	Yes
c) Quarantine delay	Yes; probability of quarantining incubating individuals in community to reflect delay of quarantine of incubating individuals in the community	yes; quarantine efficacy	Not included in the model	Yes; in term of contact tracing efficacy	Not included in the model	Yes; a progression rate from exposed state to prodrome in the disease dynamics	Yes; by a term of delay in tracing a named contact
d) Isolation delay	Yes; in terms of “probability of isolation of symptomatic individuals in the community” and “probability of isolation of symptomatic health care workers”	Yes; in terms of a distribution characterizing an individual person who has not been isolated by time since infection to reflect all individuals' infection due to this delay	Yes; proportion of the infectious period that has passed at the time the infected is isolated.	Not included in the model	Yes; 3.67 days	Yes; a progression rate from prodrome state to acute illness state in the disease dynamics	Yes; delay from symptom onset to isolation, delay from symptom onset to health seeking behaviour.
e) Quarantine efficiency	Yes; in the term of “probability of quarantining of incubating individuals in the community”	Yes; defined as quarantine efficacy	Not included in the model; assuming 100% efficiency	Yes; in term of contact tracing efficacy	Not included in the model; assuming 100% efficiency	Yes; in terms of a smaller hospitalization rate per capita of the exposed individuals	Not included in the model; assuming 100% efficiency
f) Isolation efficiency	Yes; reflected in two terms: “probability of isolation of symptomatic individuals in the community” and “probability of isolation of symptomatic health care workers”	Yes; defined as isolation efficacy and contact tracing efficiency	Not included in the model;	Not included in the model; assuming 100% efficiency	Not included in the model;	Yes; in terms of a smaller hospitalization rate per capita of the symptomatic individuals	Yes; in terms of a term of isolation effectiveness
h) Correlation structure between diseases generation due to contact tracing	No	No	No	No	No	No	Yes

#### 3.3.1 Successful Tracing Ratio of Contacts

Contacts who are successfully traced will be handled through subsequent follow-up actions such as symptom monitoring or isolation. In practical epidemiological cases, not all contacts can be successfully traced. Four studies included the factor of successful tracing ratio of contacts in their dynamics models. Peak et al. [15] and Klinkenberg et al. [17] explicitly incorporated a component for proportion of identified contacts in their models. Identified contacts were quarantined before development of symptoms. Slightly different populations targeted for quarantine were identified in our review studies. Becker et al. [21] targeted all household members and a proportion of 50% of between household contact of an infected individual. Contact tracing efficacy was defined by Chen et al. [18] to reflect this ratio. In general, quarantine of all identified contacts of diagnosed infected individuals was featured in all individual-based infection dynamics models. Ideally, identified contacts are subsequently isolated for management immediately after they have been identified through regular symptom monitor assessment as symptomatic individuals. Peak et al. further considered the fractions of traced contacts who were truly infected and defined uninfected contacts to be traced for a duration up to 95th percentile of incubation period.

#### 3.3.2 Asymptomatic Transmission Ratio

Asymptomatic transmission ratio refers to the proportion of infections that have occurred before symptom onset. Prior research showed that a significant amount of SARS Co-V transmissions were either pre-symptomatic or asymptomatic [25]. As of 18 August 2018, there has been no confirmed case caused by asymptomatic transmission for MERS, with one possible case of asymptomatic transmission occurred during the 2012 outbreak [26]. Four studies [15,[18], [19], [20]] considered this factor in their transmission models and a range of estimates from 0 to 11% were used. Feng et al. did not explicitly include this parameter in the model, but reflected the occurrence of asymptomatic transmission in infection rates contributed by exposed individuals. The study for MERS by Peak et al. assumed no asymptomatic infection [15].

#### 3.3.3 Quarantine and Isolation Delay

In practical epidemiological cases, follow-up control measures might not be implemented to contacts and infected individuals immediately after the contact event. In the 2003 SARS outbreak, it was observed that the time between symptoms onset and hospitalization was 3–5 days, with longer times earlier in the epidemic [27]. Two kinds of implementation delay were considered in the reviewed studies: quarantine delay and isolation delay. Quarantine delay refers to the time lapse between the identification of infected individuals and the implementation of quarantine to contacts. Isolation delay refers to the time lapse between symptoms onset and formal hospital diagnosis/isolation of the infected individuals.

Across the seven studies, five considered the effect of quarantine delay in the transmission dynamics using similar quantities. Peak et al. [15] specified a term called “delay in tracing a named contact”. Fraser et al. [19] used a term called “quarantine efficacy”. Lloyd Smith et al. [20] used a term called “probability of quarantining incubating individuals in community”. Chen et al. [18] used a term called “contact tracing efficacy”. Feng et al. [16] featured this delay with a progression rate from exposed state (either identified or unidentified contacts) to prodrome in the compartmental model.

Three studies characterized isolation delay in the model. Klinkenberg et al. defined a fixed value of 3.67 days between symptom onset and isolation [17]. Peak et al. [15] defined a variable value of 0–0.5 days for isolation delay, and also supplemented a uniform distributed delay from onset to health seeking behavior. Fraser et al. [19] defined a distribution characterizing an individual person who had not been isolated by the time since infection to reflect isolation delay. Other three studies did not include an explicit term for isolation delay, but reflected it in terms of other embedded quantities. Lloyd-Smith et al. [20] embedded a probability of isolating symptomatic individuals in the community and healthcare workers in their model. Feng et al. [16] reflected isolation delay with a progression rate of prodromal individuals becoming acute illness in the disease dynamics. Becker et al. [21] accounted for isolation delay with a revised proportion of the infectious period that had passed at the time the infected individuals were isolated. Chen et al. [18] did not consider the factor of isolation delay in their model.

#### 3.3.4 Quarantine and Isolation Efficiency

Contact tracing is shown to be an essential and successful public health tool in reducing the transmission risk of new emerging infectious diseases such as SARS among Hong Kong population [10]. However, the follow-up quarantine and isolation did not necessarily stop all transmission, as illustrated in nosocomial outbreaks of SARS [28] and MERS [29]. An efficiency factor is therefore sometimes incorporated in the models in order to represent the overall quarantine and isolation efficiency in transmission control. Four studies incorporated quarantine efficiency in their models. Quarantine efficacy [19], probability of quarantining individuals in the community [20], a smaller hospitalization rate per capita of the exposed individuals [16] and contact tracing efficacy [18] were used to account for quarantine efficiency. Becker et al. [21], Klinkenberg et al. [17], and Peak et al. [15] did not incorporate quarantine efficiency as a factor of their model, and assuming 100% in quarantine efficiency.

Four studies incorporated isolation efficiency as a factor in the models. Peak et al. [15] used a term named “isolation effectiveness” to represent isolation efficiency in their model. Lloyd-Smith et al. [20] reflected isolation efficiency with two terms, “probability of isolation of symptomatic individuals in the community” and “probability of isolation of symptomatic healthcare workers”. Feng et al. [16] defined a smaller hospitalization rate per capita of the symptomatic individuals (prodromal individuals or acute illness individuals) to represent isolation efficiency in their model. Fraser et al. [19] used two terms, “isolation efficacy” and “contact tracing efficiency”, to measure effectiveness of isolation for infected individuals and effectiveness of isolation for contacts respectively. Becker et al. [21], Chen et al. [18] and Klinkenberg et al. [17] did not model possible transmission that might occur while being isolated, assuming isolation to be 100% effective to stop disease transmission.

#### 3.3.5 Correlation Structure between Disease Generation Induced by Contact Tracing

Social contact structure between disease generations is affected by contact tracing and follow-up measures. Social contact is expected to be reduced when an individual is successfully traced. Out of the seven studies, only Peak et al. [15] considered this correlation structure between disease generation due to contact tracing in the model.

### 3.4 Implications of Quarantine for Controlling SARS and MERS

Quarantine is usually considered as one of the live options during disease outbreaks. Day et al. proposed a criteria for quarantine to be effective if a large number of infections were contributed by asymptomatic infection [30]. However, given the social disruption and high costs of this control measure, its poor scalability and resource constraint resulting implementation delay reduce immensely its effectiveness for epidemic containment. For SARS, Feng et al. noted that *“final outbreak size would have been smaller if greater proportion of exposed individuals being quarantined in theory but quarantine is exceedingly inefficient”* [16]. Fraser et al. concluded that “*effective isolation of symptomatic individuals is sufficient to control an outbreak for SARS*” [19]. Chen et al. concluded that “*effective isolation of symptomatic patients with low efficacy contact tracing is sufficient to control of SARS outbreak*” [18]. Peak et al. [15] found that “*comparative effectiveness of quarantine and symptom monitoring is strongly influenced by differences in the infection's natural history*”. With other intervention strategies in force, marginal benefit of quarantine was shown to be usually very low for SARS [15]. Combination of symptom monitoring with other complementary interventions, such as hand-hygiene, social distancing and protective equipment or simply isolating infected individuals only, likely controlled the diseases and ruled out the need to resort to quarantine [15,19]. For MERS, the possible substantial increase in its transmissibility resulted in difficulty in using symptom monitoring to control the disease which guaranteed the reassessment of the role of quarantine [15].

## 4 Discussion

Mathematical modelling has been a powerful tool for understanding and predicting the transmission dynamics of infectious diseases. It was particularly useful during ongoing outbreaks, and was used for evaluating public health policies for disease containment in the SARS outbreak [10], predicting transmission progression, testing efficacy and evaluating effectiveness of contact tracing during the Ebola outbreak in 2013 [31,32]. Improvements for future work and future research directions are summarized from the reviewed models.

### 4.1 Parameterization in Epidemiological Models

Tables 2 and 3 summarize the major parameters incorporated in the seven reviewed models. The two tables list eleven parameters (four key epidemiological parameters and seven intervention parameters). A primary consideration in the initial stage of constructing mathematical epidemiology models is what parameters to be included in a model. The most straightforward approach is to include as many parameters as possible to reflect disease natural history, practical intervention and control measures. A more generic and complex model incorporating all major parameters provides the flexibility of manipulating different variables to simulate outcomes under various hypothetical scenarios. However, from a practical point of view, modelers should consider whether parameters featuring disease courses and interventions can be estimated from empirical or clinical data. The availability of data for parameter estimation is the central challenge for building realistic models.

A robust and rapid estimation of parameters during the early stage of a Co—V outbreak is essential for the construction of useful models of contact tracing. When designing a mathematical epidemiology model, modelers need to ascertain what data can be collected by public health administrators, practically during an outbreak. It was not clear to us from the reports considered in this review that this was yet common practice. We recommend closed-loop communication between epidemiologists and public health administrators for assessing clinical and empirical data collection process during the initial phase of an outbreak. During that period, public health administrators should also prepare a set of operation guidelines for collecting required data.

There was considerable variation in the choice of distributions used for key elements of the natural history. Exponential distributed incubation and infectious period were normally used to ensure greater mathematical tractability. However, some studies proposed to adopt gamma distribution instead to better describe the disease stages of SARS [27,33]. Pitzer et al. [34] suggested that models with gamma distributed infectious period were best fit to the data compared with those assuming constant transmission probability or proportional relation with viral load. The nature of memory-less free characteristics of gamma distribution ensured the biology in the transmission process to be more realistic. Previous work [[35], [36], [37]] suggested the importance of realistic distribution of these quantities facilitated more understanding in the epidemic size, the disease progression and how their distributions be adjusted in response to different intervention strategies [38]. These choices are especially relevant to models of contact tracing, as was evident in the early 2000s when two apparently similar models [39,40] came to very different conclusions about likely efficacy of contact tracing because of their different implicit assumptions about the variance of the latent period distribution.

### 4.2 Social Contact Mixing Assumptions

One common assumption among the reviewed studies was homogeneity in social contact mixing. Six reviewed studies assumed homogeneous social contact mixing [[15], [16], [17], [18], [19], [20]]. This assumption was not reflecting realistic situations. Previous study found that social contact patterns was heterogeneous [41,42], and social contact pattern within age groups was a significant factor of age-specific infection rates [42]. Future models should consider contact patterns in different social settings such as home, workplace, school, hospital and community to reflect the heterogeneity in disease transmissibility.

Another common assumption among the reviewed studies was assuming social contact mixing to be the same before and after control measures were implemented. Six studies made such assumption [[16], [17], [18], [19], [20], [21]]. Fraser et al. [19] addressed this issue by pointing out this misassumption as a limitation of their model, and suggested that models incorporate this feature or alternative mechanisms [9,43] with heterogeneity transmission would give a more unbiased efficacy estimate of contact tracing. Peak et al. addressed this limitation by incorporating this factor into their model [15]. The correlation structure between infectious individuals and infected individuals for subsequent intervention measures following contact tracing should be considered.

### 4.3 Resource Constraints

All studies assumed that contact tracing and follow-up control measures were conducted with unlimited resources. None considered the practical constraints that resources for contact tracing and follow-up control measures might not be available at full throttle. Previous outbreak demonstrated that it was not feasible to have all identified contacts traced as the workforce for contact tracing were depleted in particular for top-stretched resources during the early phase of outbreaks. Public health administrators were faced with decisions and trade-offs in various resources allocation and effort prioritization along with political and social resistance. Future research questions need addressing are how many resources are required and how to prioritize the resources to contain the diseases.

## 5 Summary and Outlook

No one-size guideline is available for containment of both SARS and MERS or other emerging coronavirus. However, mathematical models can help inform policy makers by evaluating the effectiveness of different existing intervention approaches in the early phase of epidemics of new emerging and reemerging Co-V outbreak in the future. A recent confirmed case of MERS in Seoul in September 2018 [44] sparked the imminent call for epidemic counter measures preparedness. To devise appropriate decision tools for the challenges of Co-V, there are a few highlights to refine the models in future research.

First, understanding the epidemiology of the disease will be the top priority in response preparedness. It is key to ensure that proper data can be obtained for model construction during the early stage of an outbreak. To address this concern, public health authority should work closely with other government departments to work out accurate epidemiology data. Modelling researchers should also liaise with public health authority to ensure the required data can be obtained. Second, to reflect the real situation, future modelling studies should evaluate intervention tools under resource-constrained situations and how to allocate resources at different epidemic stages. Third, although SARS and MERS are both classified as coronavirus, the transmission heterogeneity, differences in relative exposure patterns [3] and practicability of control measures determine the optimal solutions for their containment. Geographic disparity of Ro estimate for SARS in 2003 due to different disease and community dependent transmission rate [45] highlighted the need for different levels of contact tracing approach during the epidemics. Forth, both hospital and community setting with the super-spreading events should be included in the transmission dynamics of Co-V outbreak to characterize the role of contact tracing in curbing the escalation of number of infections contributed by super-spreaders. Fifth, modelling approach can provide the condition that contact tracing effectively control the outbreak. Sometimes, politicians or health policy makers may hesitate to implement contact tracing. For example, SARS transmission in high-rise buildings among residents of Amoy Gardens suggested possible airborne transmission [46,47] and government officials were prone to political pressures regarding quarantine. With the help of mathematical models, they can decide when to adopt contact tracing if certain conditions are satisfied. Last but not least, model developers should incorporate factors regarding pre-symptomatic transmission and limited resources allocation against the epidemics at the beginning of the outbreak. Resources optimization and quarantine prioritization by target population will be the next step epidemiologists or modelers should focus on for the challenges of new emerging Co-V epidemics in the future. Two typical examples of the refinement of the current quarantine approach are household quarantine and quarantine based on residential geographic location. In addition to restricting movement in a particular geographic region and recommendation of infectious household members to stay home, other tracing approaches including identifying contacts at workplace or school can also be considered.

## Declarations of Interest

None.

## Acknowledgments

This work has been partially supported by Research Fund for the Control of Infectious Diseases, Hong Kong (Number: CU-17-C18, 11100642); General Research Fund (Number: 14112818); Health and Medical Research Fund (Ref: 17160302); Wellcome Trust (UK, 200861/Z/16/Z); National Institute for General Medical Sciences (US, MIDAS U01 GM110721-01); National Institute for Health Research (UK, for Health Protection Research Unit funding). The authors also thank Li Ka Shing Institute of Health Sciences for technical support.
